# Screening of a highly inhibitor-tolerant bacterial strain for 2,3-BDO and organic acid production from non-detoxified corncob acid hydrolysate

**DOI:** 10.1186/s13568-019-0879-1

**Published:** 2019-09-24

**Authors:** Jing Wu, Yu-Jie Zhou, Wen Zhang, Ke-Ke Cheng, Hong-Juan Liu, Jian-An Zhang

**Affiliations:** 1 Institute of Nuclear and New Energy Technology, Tsinghua University, Beijing, 100084 China; 2 School of Chemical Engineering and Energy Technology, Dongguan University of Technology, Dongguan, 523808 China

**Keywords:** 2,3-Butanediol, Organic acid, Atmospheric and room temperature plasma (ARTP), *Enterobacter cloacae*, Non-detoxified hydrolysate

## Abstract

Fermentation of chemicals from lignocellulose hydrolysate is an effective way to alleviate environmental and energy problems. However, fermentation inhibitors in hydrolysate and weak inhibitor tolerance of microorganisms limit its development. In this study, atmospheric and room temperature plasma mutation technology was utilized to generate mutant strains of *Enterobacter cloacae* and screen for mutants with high inhibitor tolerance to acid hydrolysate of corncobs. A highly inhibitor-tolerant strain, *Enterobacter cloacae* M22, was obtained after fermentation with non-detoxified hydrolysate, and this strain produced 24.32 g/L 2,3-butanediol and 14.93 g/L organic acids. Compared with that of the wild-type strain, inhibitor tolerance was enhanced twofold with M22, resulting in improvement of 2,3-butanediol and organic acid production by 114% and 90%, respectively. This work presents an efficient method to screen for highly inhibitor-tolerant strains and evidence of a novel strain that can produce 2,3-butanediol and organic acids using non-detoxified acid hydrolysate of corncobs.

## Introduction

2,3-Butanediol (2,3-BDO) is a staple chemical material and liquid fuel that is used extensively in the medical, food, and aerospace industries. 2,3-BDO can be used to manufacture flavour agents, edible spices, and solvents (Garg and Jain 1995; Tran and Chambers 1987; Zeng et al. 2016). The traditional chemical method used tetracarbonic hydrocarbons produced by petroleum pyrolysis as raw material and then obtained 2,3-butanediol by hydrolysis at high temperature and pressure. Dependence on petroleum resources and high production costs have limited the development of chemical method. Biological fermentation is not dependent on oil resources and therefore is considered a green route for 2,3-BDO production, with carbohydrates converted to 2,3-BDO by microorganisms under mild conditions. Fermentation is accompanied by the formation of organic acids such as formic acid, acetic acid, and succinic acid, which also have many applications. In recent years, succinic acid has been extensively used in the synthesis of biodegradable polymers such as polybutylene succinate (PBS) and polyamide (Debuissy et al. 2017; Jiang et al. 2014).

The feedstock for 2,3-BDO and organic acid fermentation has been a focus of study worldwide. Typical 2,3-BDO fermentation used sugars such as glucose and xylose as substrates. Because of the high cost of sugars, some cheap feedstock such as starch, molasses, glycerol, and lignocellulosic materials have been utilized as substrates for 2,3-BDO fermentation (Bialkowska and Aneta 2016). Lignocellulosic materials are considered the most plentiful feedstock and an alternative for 2,3-BDO and organic acid production. The hemicellulose fraction accounts for 20–35% of lignocellulose materials, and hemicellulose is prone to being hydrolysed into monosaccharides with diluted acid pretreatment. However, the formation of by-products (e.g. formic acid, acetic acid, furfural, and 5-hydroxymethyl furfural) always accompanies dilute acid pretreatment of lignocellulose, which inhibits microbial growth and fermentation (Lloyd and Wyman 2005). Therefore, these inhibitors need to be removed when using dilute acid hydrolysate of lignocellulose as a substrate for 2,3-BDO fermentation. Many processes such as activated carbon adsorption, calcium neutralization, and organic solvent extraction have been applied to detoxification of the dilute acid hydrolysate of lignocellulose (Cheng et al. 2010; Jiang et al. 2012). However, the process of detoxification often leads to the loss of sugar, which reduces production yield. Another disadvantage of this process is the consumption of additional reagents that cannot be recycled.

The high raw material and operating costs of hydrolysate detoxification make fermentation uneconomical. Thus, using non-detoxified acid hydrolysate as a raw material for fermentation with inhibitor-tolerant strains seems attractive (Schirmer-Michel et al. 2009; Varga et al. 2004). Most wild-type microbes have low inhibitor tolerance. Mutation breeding methods are effective in improving strain characteristics. Many physical and chemical mutation methods involving ultraviolet radiation, X-ray, sodium azide, and diethyl sulphate have been reported in the literature (Bhagwat and Duncan 1998; Wang et al. 2007). However, traditional mutation methods are detrimental to health and have low mutation efficiency.

Atmospheric and room temperature plasma (ARTP) mutation is a novel technique that can be applied to the breeding of bacteria, fungi, yeast, actinomycetes, and microalgae (Zhang et al. 2014a), is easy and safe to perform, and has high mutation efficiency. Wang et al. used ARTP to mutate *Streptomyces avermitilis* and obtained a mutant with a higher abamectin yield (Wang et al. 2010). ARTP mutation was also applied to the breeding of *Crypthecodinium cohnii* for extracellular polysaccharide production (Liu et al. 2015). Cao et al. used the oleaginous microalgae *Chlorella pyrenoidosa* as the parent strain and, by ARTP, obtained a mutant with high lipid productivity. The dry weight and lipid productivity of the mutant strain were increased by 22.07% and 16.85%, respectively (Cao et al. 2017). These results confirm that ARTP is an efficient mutagenesis tool for microbial breeding.

Many microorganisms such as *Enterobacter*, *Klebsiella*, *Bacillus*, *Aeromonas*, *Pseudomonas*, *Serratia* have been employed for 2,3-BDO production (Garg and Jain 1995; Bialkowska 2016)*. Enterobacter cloacae* is considered to be one of the most promising strain for industrial application because of its stable performance under a wide range of environmental conditions and prominent tolerance to inhibitors. In addition, *Enterobacter cloacae* can use an extensive range of substrates to produce 2,3-BDO. The ability of *Enterobacter cloacae* to produce 2,3-BDO and succinic acid have been investigated by our group. *Enterobacter cloacae* can produce 77.1 g/L 2,3-BDO and 28.1 g/L succinic acid from glucose. When using xylose as substrate, a maximum of 40.67 g/L 2,3-BDO and 21.79 g/L succinic acid were obtained (Cheng et al. 2013; Wu et al. 2018). So, *Enterobacter cloacae* was used as the original strain in this study. *Enterobacter cloacae* cannot grow at the original pH of hydrolysate which is about 2. Appropriate pH of cell growth and 2,3-BDO formation is 6.5. So the tolerance of *Enterobacter cloacae* to hydrolysate in this study was conducted at pH 6.5.

In this study, the ARTP mutation technique was used to create a library of *Enterobacter cloacae* mutants to screen for strains with high tolerance to inhibitors in corncob acid hydrolysate. The mutant strains were then used to produce 2,3-BDO and organic acids with non-detoxified corncob acid hydrolysate as the substrate. The selected mutant strain may be applied to biological detoxification of dilute acid hydrolysate of lignocellulose in other fermentation processes.

## Materials and methods

### Strain and media

The original strain used in this study was *E. cloacae* CICC 10011, which was purchased from the China Center of Industrial Culture Collection (CICC). The *E. cloacae* M22 obtained in this study has been deposited in China General Microbiological Culture Collection Center (CGMCC) and the preservation number is CGMCC NO. 17265.

The seed medium consisted of 30 g/L xylose, 2 g/L (NH_4_)_2_SO_4_, 4.4 g/L K_2_HPO_4_, 1.3 g/L KH_2_PO_4_, 1 g/L yeast extract, 0.2 g/L MgSO_4_·7H_2_O, 1 mL/L trace element solution, and 2 mL/L Fe solution. The trace element solution contained 70 mg/L ZnCl_2_, 0.1 g/L MnCl_2_·4H_2_O, 60 mg/L H_3_BO_3_, 0.2 g/L CoCl_2_·2H_2_O, 20 mg/L CuCl_2_·2H_2_O, 25 mg/L NiCl_2_·6H_2_O, 35 mg/L Na_2_MoO_4_·2H_2_O, 0.9 mL/L HCl, and Fe solution containing 5 g/L FeSO_4_·7H_2_O, and 4 mL/L HCl. The pH of seed medium was 6.5.

Acid hydrolysate of corncobs was diluted to various concentrations and used as fermentation medium containing 1.5 g/L yeast extract.

### Corncob pretreatment

The corncobs used as raw material for hydrolysis of dilute acids was obtained from Shandong province in China. The particle size of the corncobs was 20–40 mesh, and the main components of corncob are 2.67 ± 0.04% benzene-ethanol extractives, 40.46 ± 0.45% cellulose, 32.87 ± 0.48% hemicellulose, 17.12 ± 0.34% lignin.

Corncob particles were pre-treated with a mixed acid solution (1.5% sulfuric acid + 0.5% phosphoric acid), loaded into 500-mL flasks with a loading capacity of 30 g and 90 g, respectively, and then heated to 130 °C for 1 h in an autoclave. The hydrolysate and solid residues were separated by filtration, and the filter cake was washed with 60 mL distilled water to increase the amount of sugars collected. The pH of the hydrolysate was adjusted to 5.5 with NaOH and 6.5 with ammonia.

### Tolerance of *Enterobacter cloacae* wild-type strains to hydrolysate

Seed culture and fermentation experiments were carried out in a 500-mL flask. The strains were inoculated into 50 mL seed culture medium, and the flasks were cultivated on a shaker at 30 °C and 150 rpm for 14–16 h. Then, the seeds were inoculated into fermentation medium containing 25%, 50%, 75%, and 100% corncob acid hydrolysate at an inoculation rate of 10% and cultivated on a shaker at 35 °C and 150 rpm for fermentation. CaCO_3_ (5 g/L) was used as a pH buffer during fermentation.

### Atmospheric and room temperature plasma mutation of *Enterobacter cloacae*

**Fig. 1:**
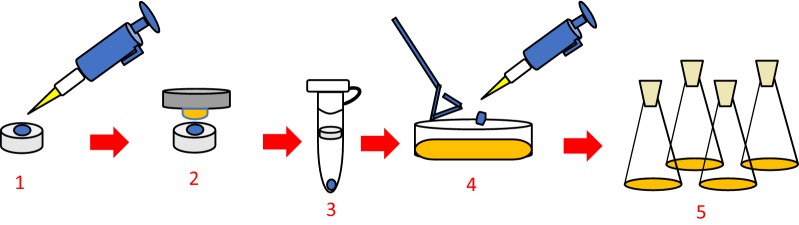
ARTP mutation process (1. Sample loading. 2. Plasma treatment. 3. Bacterial elution. 4. Plate screening. 5. Fermentation screening)

The ARTP mutation breeding system was supplied by Professor Xinhui Xing of Tsinghua University and consisted of a coaxial type plasma generator, gas supply control subsystem, radiofrequency power supply, and sample plate made of stainless steel (Zhang et al. 2014b). The experimental process is shown in Fig. 1. Bacteria reached logarithmic growth (OD ≈ 1) before mutation. Ten microliters of the bacterial liquid was pipetted onto a sterilized sample plate and then treated for 0, 60, 120, 180, 240, 300, or 360 s. The helium gas flow was 10 standard litres per minute, and plasma was induced at 120 W.

After ARTP mutation, the cells on the stainless-steel plates were washed with fresh seed culture medium and then cultivated on a shaker at 30 °C and 150 rpm for 1 h. The activated bacteria were properly diluted, and 100 μL bacterial suspension was coated on each plate with 75% diluted acid hydrolysate. The plates were placed in an incubator for 24 h at 30 °C, and then the numbers of surviving clones were counted.

### Fermentation experiments

The seed cells were inoculated into 500-mL flasks containing 100 mL fermentation medium at 10% (v/v), then cultivated on a shaker at 35 °C and 150 rpm for fermentation. The pH was buffered with 5 g/L GaCO_3_. Each data point is the average of two replicates.

Fermentation was performed in a 1-L fermenter at 35 °C and 500 rpm with ventilation of 0.2 vvm. The seed culture was transferred to 500 mL fermentation medium with an inoculation volume of 10%, and the pH was maintained at 6.5 throughout fermentation by adding 4 mol/L sodium hydroxide solution with an automatic controller.

### Analytical methods

Cell concentration was monitored by measuring the optical density at 600 nm (OD_600_) with a UV spectrophotometer (JingHua 7600CRT, China). Then, components of the hydrolysate and fermentation products were analysed using a high-performance liquid chromatograph (HPLC, Shimadzu LC-20AT, Japan). The components were separated in an Aminex HPX-87H ion exclusion column (Bio Rad, USA) at 65 °C, and 5 mM H_2_SO_4_ was used as the mobile phase with a flow rate of 0.8 mL/min. Concentrations of glucose, xylose, formic acid, acetic acid, succinic acid, and 2,3-BDO were determined using an RI detector. HMF and furfural were analysed with a UV detector at 210 nm.

## Results

### Corncob pretreatment

Table 1 shows the main compounds of the dilute sulphuric acid hydrolysate from corncobs. The concentration of total sugars was 67.57 g/L, and the mass ratio of glucose, xylose, and arabinose was 1:8:1.2. Xylose was the main carbon source and was attributable to the degradation of hemicellulose during dilute acid pretreatment. The amount of acetic acid was greater than that of the other inhibitors.

**Table 1:** Main components of dilute acid hydrolysate from corncobs

Glucose (g/L)	Xylose (g/L)	Arabinose (g/L)	Formic acid (g/L)	Acetic acid (g/L)	HMF (g/L)	Furfural (g/L)
6.66 ± 0.13	52.79 ± 1.02	8.12 ± 0.22	0.76 ± 0.03	7.26 ± 0.31	0.24 ± 0.01	0.89 ± 0.10

### Tolerance of *Enterobacter cloacae* wild-type strains to hydrolysate

**Fig. 2:**
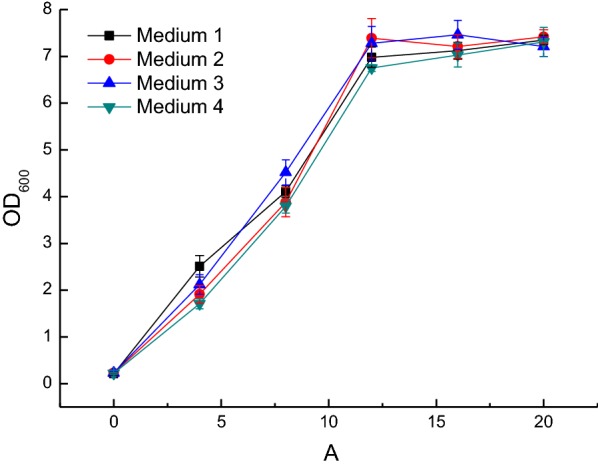
Cell growth in different sugar concentrations (Medium 1: 1.5 g/L glucose, 12 g/L xylose, 2 g/L arabinose. Medium 2: 3 g/L glucose, 24 g/L xylose, 3.5 g/L arabinose. Medium 3: 4.5 g/L glucose, 36 g/L xylose, 5.5 g/L arabinose. Medium 4: 6 g/L glucose, 48 g/L xylose, 7 g/L arabinose.)

Culture medium containing 25%, 50%, 75%, or 100% acid hydrolysate from corncobs was used as a substrate to investigate the tolerance of *E. cloacae* to hydrolysate. In addition, culture medium containing same sugar concentration as hydrolysate were used to study the effects of different sugar concentrations on cell growth. The results showed that sugar concentration had no significant effect on cell growth (Fig. 2), which proved that the sugars in hydrolysate have no inhibitory effect on cell growth within the range of concentration investigated. Tables 2 and 3 show the concentration of each component before and after fermentation, respectively. Use of 25% hydrolysate resulted in no obvious inhibition of *E. cloacae.* The sugar consumption achieved 99.01% at 18 h with a sugar consumption rate of 0.83 g/L/h. The final concentration of 2,3-BDO was 6.27 g/L. The 2,3-BDO productivity and yield were 0.35 g/L/h and 0.42 g/g sugar, respectively. The initial concentrations of formic acid and acetic acid were 0.17 g/L and 1.68 g/L, respectively, in 25% hydrolysate. The production of organic acids during fermentation were 0.82 g/L succinic acid, 0.04 g/L formic acid and 3.05 g/L acetic acid. When 50% hydrolysate was used as the substrate, the utilization of sugar decreased to 85% and the fermentation time was extended to 40 h due to relatively high inhibition. The sugar consumption rate was only 0.69 g/L/h. The production of 2,3-BDO increased to 10.84 g/L but the the 2,3-BDO productivity and yield decreased to 0.27 g/L/h and 0.39 g/g sugar, respectively. The values of organic acids in Table 3 were the final concentrations in the fermentation broth. The actual amount of organic acids formed during fermentation were 1.43 g/L succinic acid, 0.02 g/L formic acid and 5.47 g/L acetic acid. Use of 75% hydrolysate almost completely inhibited the growth of *E. cloacae*. Only 2% of sugar was utilized, and cell growth only reached 0.53. Therefore, 75% hydrolysate was used as the preliminary screening medium.

**Table 2:** The concentration of each component before fermentation

Hydrolysate concentration (%)	Glucose (g/L)	Xylose (g/L)	Arabinose (g/L)	Formic acid (g/L)	Acetic acid (g/L)	HMF (g/L)	Furfural (g/L)
25	1.11 ± 0.05	12.17 ± 0.17	1.82 ± 0.03	0.17 ± 0.01	1.68 ± 0.15	0.05 ± 0.01	0.21 ± 0.02
50	2.98 ± 0.10	25.86 ± 0.88	3.59 ± 0.11	0.34 ± 0.01	3.22 ± 0.12	0.10 ± 0.01	0.39 ± 0.02
75	4.56 ± 0.12	35.77 ± 1.03	5.53 ± 0.12	0.52 ± 0.03	5.03 ± 0.16	0.17 ± 0.02	0.61 ± 0.01
100	5.72 ± 0.09	47.63 ± 1.15	7.09 ± 0.20	0.68 ± 0.02	6.41 ± 0.15	0.21 ± 0.02	0.79 ± 0.03

**Table 3:** Results of fermentation with different concentrations of hydrolysate

Hydrolysate concentration (%)	Sugar utilization (%)	Succinic acid (g/L)	Formic acid (g/L)	Acetic acid (g/L)	Acetoin (g/L)	2,3-BDO (g/L)	Ethanol (g/L)	OD_600_
25	99.01 ± 0.25	0.82 ± 0.08	0.21 ± 0.02	4.73 ± 0.17	0.89 ± 0.10	6.27 ± 0.15	0.22 ± 0.04	6.78 ± 0.11
50	85.12 ± 0.62	1.43 ± 0.12	0.36 ± 0.04	8.69 ± 0.21	1.02 ± 0.08	10.84 ± 0.26	0.37 ± 0.07	6.66 ± 0.20
75	2.03 ± 0.11	–	0.49 ± 0.01	5.51 ± 0.13	–	–		0.53 ± 0.06
100	–	–	0.66 ± 0.02	6.34 ± 0.32	–	–		–

### Mutation and primary screening of the mutants

**Fig. 3:**
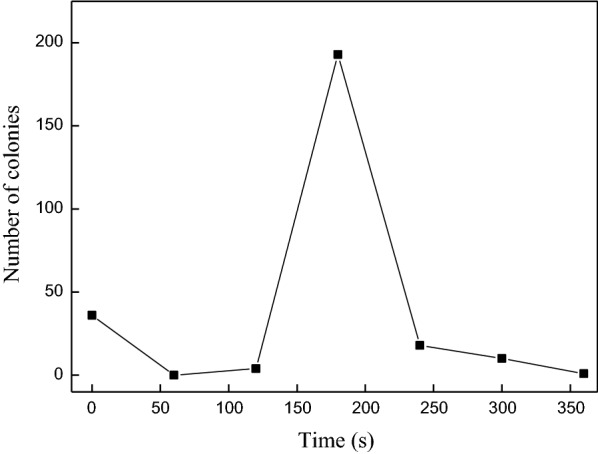
Number of colonies on plates under different mutation times

The mutants were screened by culture and fermentation, and, in general, a lethality rate of 90–99% was considered appropriate. In this study, an efficient and rapid screening method of directional screening on plates containing 75% diluted acid hydrolysate was developed. The number of colonies on plates after different treatment times is shown in Fig. 3. *E. cloacae* was subjected to ARTP mutation for times ranging from 60 to 360 s. Untreated *E.* cloacae, used as a control, resulted in 30 surviving clones. The corresponding numbers of surviving colonies were 0, 4, 193, 18, 10, and 1 when the treatment times were 60, 120, 180, 240, 300, and 360 s, respectively. When the treatment time was less than 180 s, the positive mutation rate was low and few colonies grow on the plates due to the presence of inhibitors. The positive mutation rate reached the maximum at 180 s of treatment time and the number of colonies on the plate increased sharply. When the treatment time was greater than 180 s, most of the cells died because of the high lethal rate, and few colonies grew on the plate. Therefore, an ARTP mutation time of 180 s was considered optimal. One hundred and ninety-three colonies were selected from the solid medium and inoculated into test tubes containing 5 mL seed culture medium for 14 h incubation at 30 °C. Then, the bacterial fluid was streak-inoculated onto plates containing 100% hydrolysate medium. There were only 23 strains that grew on the 100% hydrolysate solid medium.

Treatment time is a key parameter in ARTP mutation. The optimum treatment time is determined by the original strain and screening conditions. For example, 60 s of treatment time is suitable for the mutation of *Spirulina platensis* to improve carbohydrate productivity (Fang et al. 2013), whereas Hua used LB agar plates containing 9.0% NaCl to screen for mutants of *E. cloacae.* At a treatment time of 2 min, 90% cell lethality was observed (Hua et al. 2010). As for the oleaginous microalgae *Chlorella pyrenoidosa,* when the treatment time was 40 s, 50 s, or 60 s, mortality rates reached 95.69%, 98.05%, and 99.88%, respectively (Cao et al. 2017).

### Fermentation of 2,3-butanediol and organic acids by mutants with hydrolysate

**Fig. 4:**
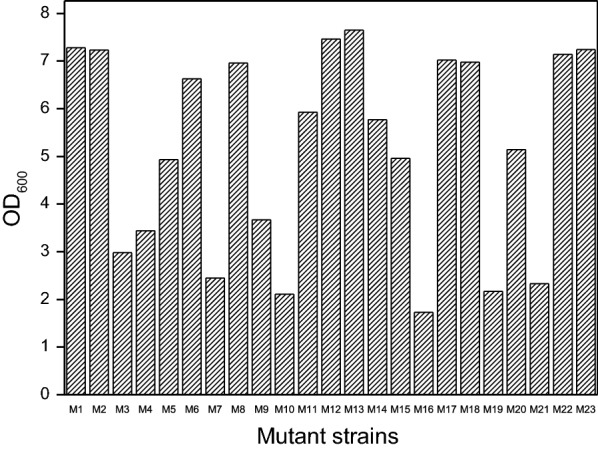
Cell growth of 23 mutants in 100% hydrolysate

The fermentation performance of 23 mutants was investigated with 100% hydrolysate as the substrate. Figure 4 shows the cell growth of mutants at 24 h. Only 10 mutants were able to grow steadily (OD_600_ > 6.0) and produce 2,3-BDO and organic acids. Another 13 mutants grew to varying degrees, but there were no fermentation products. Table 4 shows the fermentation results for the mutants. The sugar utilization of 10 mutants was higher than 80%, and their ability to ferment 2,3-BDO and organic acids with non-detoxified hydrolysate was much greater than that of the wild-type strain. Good genetic stability is essential for a mutant to be optimal for fermentation. Therefore, the stable fermentation performance of the mutants with non-detoxified hydrolysate was verified. The mutants were cultivated in seed medium for five generations and then inoculated into non-detoxified hydrolysate for fermentation. The inhibitor tolerance of most mutants decreased, with only mutant M22 maintaining greater fermentation ability (Table 5). The sugar utilization of M22 was 88.69%, and the production of 2,3-BDO and organic acids was 24.32 g/L and 14.93 g/L, respectively. Long-term stability of M22 is shown in Table 6. The 43 generations of M22 transmission still maintained good fermentation performance.

**Table 4:** Results of fermentation with non-detoxified hydrolysate

Mutants	Sugar utilization (%)	Succinic acid (g/L)	Formic acid (g/L)	Acetic acid (g/L)	Acetoin (g/L)	2,3-BDO (g/L)	Ethanol (g/L)	OD_600_
*M*1	87.04 ± 1.51	2.70 ± 0.26	1.52 ± 0.10	6.98 ± 0.26	1.04 ± 0.12	14.62 ± 1.03	1.71 ± 0.21	7.28 ± 0.33
*M*2	86.71 ± 0.98	2.49 ± 0.17	1.92 ± 0.13	6.73 ± 0.30	0.87 ± 0.07	16.48 ± 1.15	1.79 ± 0.14	7.23 ± 0.21
*M6*	84.88 ± 1.22	1.24 ± 0.05	1.05 ± 0.08	8.08 ± 0.22	3.18 ± 0.23	15.29 ± 0.77	1.02 ± 0.10	6.63 ± 0.16
*M8*	85.20 ± 1.53	1.48 ± 0.22	1.28 ± 0.01	7.01 ± 0.29	1.96 ± 0.16	14.60 ± 0.53	1.15 ± 0.07	6.69 ± 0.20
*M12*	85.67 ± 2.01	1.87 ± 0.14	0.72 ± 0.06	6.77 ± 0.37	1.98 ± 0.09	15.23 ± 0.46	0.87 ± 0.11	7.46 ± 0.13
*M13*	85.39 ± 1.10	1.73 ± 0.20	1.34 ± 0.12	6.65 ± 0.18	1.84 ± 0.14	14.74 ± 0.82	0.86 ± 0.04	7.65 ± 0.17
*M1*7	87.67 ± 2.57	1.86 ± 0.11	1.27 ± 0.08	6.90 ± 0.21	1.71 ± 0.21	15.29 ± 0.65	1.21 ± 0.10	7.02 ± 0.25
*M1*8	85.46 ± 1.72	1.69 ± 0.14	2.18 ± 0.15	6.76 ± 0.13	1.80 ± 0.06	14.85 ± 0.19	1.11 ± 0.02	6.98 ± 0.19
*M22*	85.98 ± 1.69	1.52 ± 0.09	1.15 ± 0.08	7.01 ± 0.09	2.21 ± 0.10	14.49 ± 0.29	0.88 ± 0.04	7.14 ± 0.08
*M23*	85.58 ± 2.41	1.70 ± 0.12	1.38 ± 0.11	6.77 ± 0.26	1.71 ± 0.20	14.90 ± 0.40	1.18 ± 0.12	7.24 ± 0.20

**Table 5:** Fermentation stability of mutants with non-detoxified hydrolysate

Mutants	Sugar utilization (%)	Succinic acid (g/L)	Formic acid (g/L)	Acetic acid (g/L)	Acetoin (g/L)	2,3-BDO (g/L)	Ethanol (g/L)	OD_600_
*M*1	–	–	0.72 ± 0.06	7.84 ± 0.28	–	–	–	–
*M*2	39.05 ± 0.69	0.79 ± 0.04	1.28 ± 0.11	6.19 ± 0.19	2.18 ± 0.12	14.01 ± 0.64	1.01 ± 0.02	7.33 ± 0.20
*M6*	12.17 ± 0.33	–	1.34 ± 0.09	7.70 ± 0.37	2.01 ± 0.12	7.10 ± 0.35	–	7.14 ± 0.18
*M8*	–	–	0.69 ± 0.01	7.69 ± 0.24	–	–	–	–
*M12*	–	–	0.68 ± 0.03	7.98 ± 0.31	–	–	–	–
*M13*	3.80 ± 0.19	–	0.69 ± 0.01	7.45 ± 0.29	–	–	–	5.96 ± 0.08
*M1*7	–	–	0.70 ± 0.02	7.17 ± 0.30	–	–	–	–
*M1*8	–	–	0.68 ± 0.03	7.56 ± 0.11	–	–	–	–
*M22*	88.69 ± 1.09	1.80 ± 0.09	2.18 ± 0.11	10.95 ± 0.76	2.72 ± 0.07	24.32 ± 0.71	1.27 ± 0.02	7.11 ± 0.12
*M23*	18.36 ± 0.59	0.46 ± 0.06	1.37 ± 0.07	8.01 ± 0.15	2.29 ± 0.11	9.53 ± 0.22	–	6.89 ± 0.19

**Table 6:** Fermentation stability of *E. aerogenes* M22 with non-detoxified hydrolysate

Generation	Sugar utilization (%)	Succinic acid (g/L)	Formic acid (g/L)	Acetic acid (g/L)	Acetoin (g/L)	2,3-BDO (g/L)	Ethanol (g/L)	OD_600_
12	87.12 ± 0.99	1.69 ± 0.11	2.03 ± 0.10	9.92 ± 0.53	2.61 ± 0.15	23.59 ± 0.82	1.11 ± 0.07	7.23 ± 0.12
23	88.63 ± 1.04	1.75 ± 0.09	2.41 ± 0.17	11.07 ± 0.21	3.03 ± 0.12	24.71 ± 0.33	1.44 ± 0.04	6.99 ± 0.25
31^a^	97.15 ± 1.25	1.44 ± 0.10	2.01 ± 0.06	8.97 ± 0.18	2.68 ± 0.13	19.99 ± 0.61	1.19 ± 0.07	7.14 ± 0.13
40^a^	96.71 ± 0.78	1.34 ± 0.06	1.93 ± 0.09	8.74 ± 0.22	2.61 ± 0.11	20.07 ± 0.55	1.02 ± 0.06	7.01 ± 0.26
43	87.73 ± 1.12	1.78 ± 0.20	2.11 ± 0.06	10.33 ± 0.19	2.74 ± 0.13	23.87 ± 0.57	1.23 ± 0.03	7.12 ± 0.22

^a^The substrate was 75% hydrolysate

**Fig. 5:**
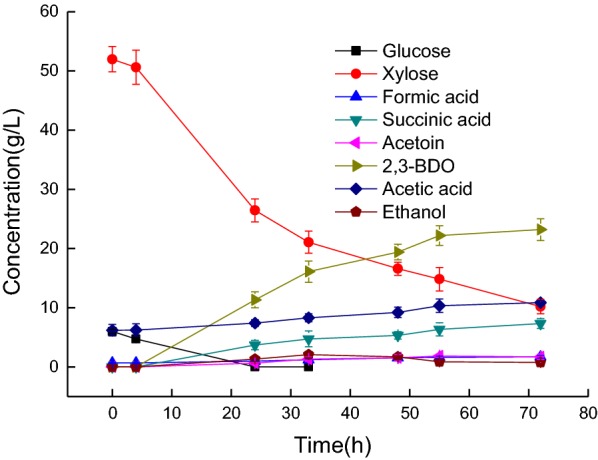
Co-production of 2,3-BDO and organic acids by M22 with non-detoxified hydrolysate

M22 was used for the co-production of 2,3-BDO and organic acids with non-detoxified hydrolysate in a 1-L fermenter. Sodium bicarbonate (5 g/L) was added at fermentation times of 12 and 36 h, respectively, to enhance the production of succinic acid. Previously, *E. cloacae* has been reported to show high co-production of 2,3-BDO and succinic acid from sugar (Cheng et al. 2013; Wu et al. 2018), and addition of bicarbonate salts to the fermentation medium can increase the amount of dissolved CO_2_, which is an indispensable substrate for the formation of succinic acid. The final concentration of succinic acid reached 7.33 g/L in this study. After fermentation, 1.02 g/L formic acid and 4.68 g/L acetic acid were produced. The final concentrations of 2,3-BDO and organic acids were 23.2 g/L and 19.93 g/L, respectively, the yield of 2,3-BDO was 0.44 g/g sugar. (Figure 5).

## Discussion

The inhibition of fermentation inhibitors in acid hydrolysate on microorganisms limit the utilization of lignocellulose material. The mechanisms underlying inhibition of organic acids and furans have been reported. Undissociated acetic acid is the key factor for inhibition of other microorganisms (Cheng et al. 2005). Molecules of organic acids in a free state can freely shuttle through cell membranes, leading to acidification of the intracellular environment and inhibiting the synthesis of DNA and RNA (Parawira and Tekere 2011). In 2,3-BDO fermentation, a small amount of acetic acid has no obvious inhibitory effect; rather, it is beneficial (Wu et al. 2013a). However, the simultaneous presence of acetic acid and furfural strongly inhibits interactions (Wu et al. 2013b). Furan derivatives, mainly furfural and HMF, are dehydrated from pentose and hexose, respectively, in acidic environments. Their inhibitory effects on microorganisms are as follows: (i) they destroy the integrity of protoplasmic membranes and increase the permeability of cell membranes; (ii) they have direct inhibitory effects on various enzymes in the glycolysis pathway; (iii) they inhibit the synthesis or accelerate the degradation of NAD(P)H in cells; and (iv) dipole moments of large aldehyde groups result in a sharp rise in reactive oxygen in cells, which damages the cytoskeleton and inhibits cell growth (Allen et al. 2010; Feron et al. 1991; Liu et al. 2013; Miller et al. 2009). According to the literature, the furfural has higher negative effect of on biomass formation. The inhibitions of HMF and acetic acid were about 1/2 and 1/10, respectively, of that of furfural (Wu et al. 2013a). In this study, the furfural and HMF were converted to their less toxic alcohols, furfuryl alcohol and furan-2,5-diyldimethanol, respectively, during the fermentation. Phenolic compounds in hydrolysate are derived from lignin as it degrades. These compounds can destroy or decompose cytomembranes. In general, the smaller the substituents on aromatic rings, the greater the inhibition (Almeida 2010).

Fermentation of 2,3-BDO from non-detoxified biomass hydrolysate by *Enterobacter* has been reported previously (Table 7). Hazeena et al. used non-detoxified oil palm frond hydrolysate as the substrate for fermentation of 2,3-BDO by *E. cloacae* SG-1. The lag period of bacterial growth was longer than that of glucose because of inhibitors present in the hydrolysate, and the concentration of 2,3-BDO was 7.67 g/L (Hazeena et al. 2016). Joo et al. investigated the effects of inhibitory compounds on *E. aerogenes.* Three hydrolysates, yellow poplar, larix, and rice hull hydrolysates, were used as substrates to produce 2,3-BDO, and 14.27 g/L, 12.44 g/L, and 10.24 g/L, respectively, were produced (Joo et al. 2016). *E. aerogenes* ATCC 29007 also has the ability to resist inhibitors. With a Miscanthus hydrolysate containing 34.62 g/L of reducing sugar as a substrate, 11.00 g/L of 2,3-BDO was obtained (Lee et al. 2015).

**Table 7:** Fermentation of 2,3-BDO from undetoxified biomass hydrolysate

Raw material	Strain	2,3-BDO (g/L)	Reference
Oil palm frond	*E. cloacae* SG-1	7.67	Hazeena et al. (2016)
Yellow poplar	*E. aerogenes* KCTC 2190	14.27	Joo et al. (2016)
Larix		12.44	
Rice hull		10.24	
Miscanthus	*E. aerogenes* ATCC 29007	11.00	Lee et al. (2015)
Corncob	*E. cloacae* CICC 10011	10.84	This study
Corncob	*E. cloacae* M22	23.20	This study

In this study, ARTP mutation technology was used to generate mutants of *E. cloacae* to screen for a strain with high inhibitor tolerance to corncob acid hydrolysate. Fermentation performance and genetic stability with non-detoxified hydrolysate of one mutant, *E. cloacae* M22, was examined. Compared with that of the wild-type strain, inhibitor tolerance was enhanced twofold in M22. The wild-type strains can tolerate only 50% hydrolysate and produce 10.84 g/L 2,3-BOD and 6.92 g/L organic acids. The mutant strain M4 can tolerate 100% hydrolysate and produce 23.2 g/L 2,3-BOD and 13.03 g/L organic acids which increased by 114% and 88%, respectively. *E. cloacae* M22 showed super inhibitor tolerance, and 23.2 g/L 2,3-BDO was produced from non-detoxified corncob acid hydrolysate. This production yield was superior to that reported elsewhere (Hazeena et al. 2016; Joo et al. 2016; Lee et al. 2015).
